# Nano delivery of simvastatin targets liver sinusoidal endothelial cells to remodel tumor microenvironment for hepatocellular carcinoma

**DOI:** 10.1186/s12951-021-01205-8

**Published:** 2022-01-04

**Authors:** Zhuo Yu, Jianfeng Guo, Yun Liu, Menglin Wang, Zhengsheng Liu, Yueqiu Gao, Leaf Huang

**Affiliations:** 1 Division of Pharmacoengineering and Molecular Pharmaceutics, Eshelman School of Pharmacy, University of North Carolina, Chapel Hill, NC 27599 USA; 2 Department of Liver Disease, Shuguang Hospital, Affiliated to Shanghai University of Traditional Chinese Medicine, Shanghai, 201203 China; 3 School of Pharmaceutical Sciences, Jilin University, Changchun, 130021 China

**Keywords:** Liver sinusoidal endothelial cells, Hepatocellular carcinoma, Simvastatin, Nanoparticles, Tumor microenvironment remodeling

## Abstract

**Background:**

Hepatocellular carcinoma (HCC) developed in fibrotic liver does not respond well to immunotherapy, mainly due to the stromal microenvironment and the fibrosis-related immunosuppressive factors. The characteristic of liver sinusoidal endothelial cells (LSECs) in contributing to fibrosis and orchestrating immune response is responsible for the refractory to targeted therapy or immunotherapy of HCC. We aim to seek a new strategy for HCC treatment based on an old drug simvastatin which shows protecting effect on LSEC.

**Method:**

The features of LSECs in mouse fibrotic HCC model and human HCC patients were identified by immunofluorescence and scanning electron microscopy. The effect of simvastatin on LSECs and hepatic stellate cells (HSCs) was examined by immunoblotting, quantitative RT-PCR and RNA-seq. LSEC-targeted delivery of simvastatin was designed using nanotechnology. The anti-HCC effect and toxicity of the nano-drug was evaluated in both intra-hepatic and hemi-splenic inoculated mouse fibrotic HCC model.

**Results:**

LSEC capillarization is associated with fibrotic HCC progression and poor survival in both murine HCC model and HCC patients. We further found simvastatin restores the quiescence of activated hepatic stellate cells (aHSCs) via stimulation of KLF2-NO signaling in LSECs, and up-regulates the expression of CXCL16 in LSECs. In intrahepatic inoculated fibrotic HCC mouse model, LSEC-targeted nano-delivery of simvastatin not only alleviates LSEC capillarization to regress the stromal microenvironment, but also recruits natural killer T (NKT) cells through CXCL16 to suppress tumor progression. Together with anti-programmed death-1-ligand-1 (anti-PD-L1) antibody, targeted-delivery of simvastatin achieves an improved therapeutic effect in hemi-splenic inoculated advanced-stage HCC model.

**Conclusions:**

These findings reveal an immune-based therapeutic mechanism of simvastatin for remodeling immunosuppressive tumor microenvironment, therefore providing a novel strategy in treating HCC.

**Graphical Abstract:**

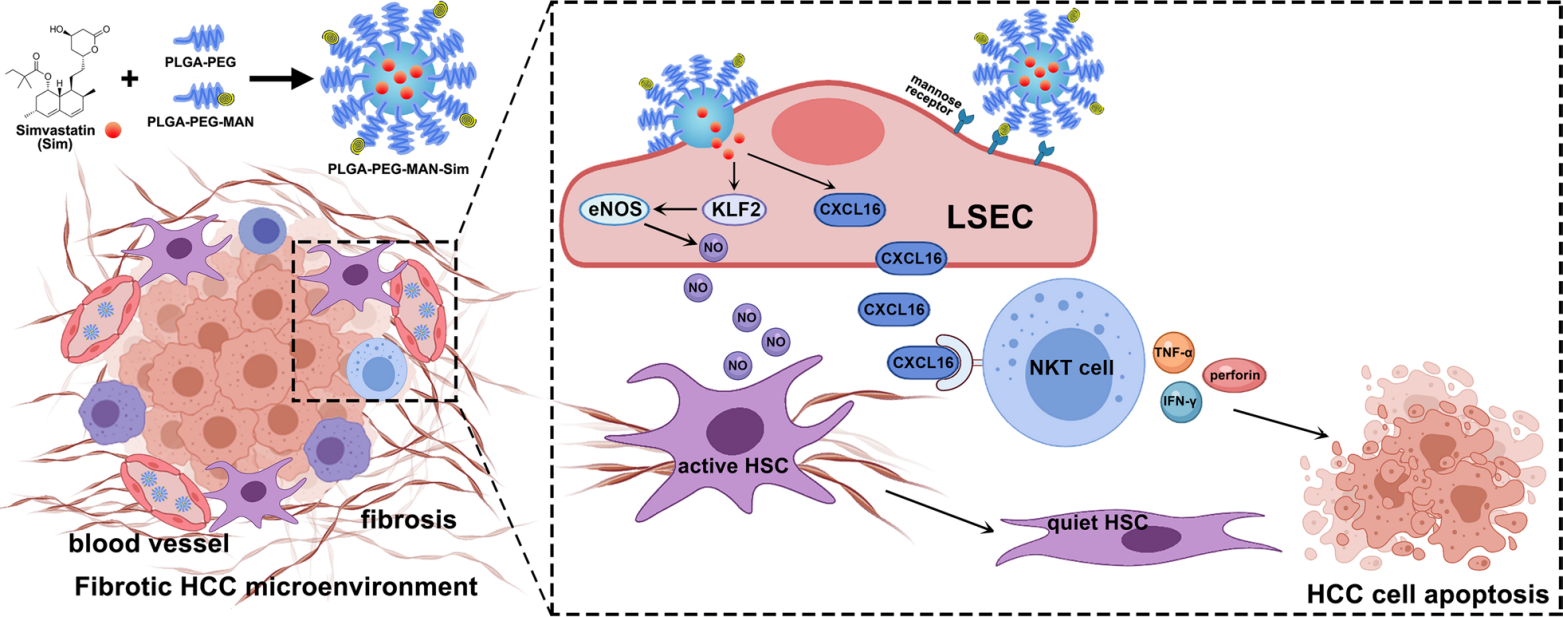

**Supplementary Information:**

The online version contains supplementary material available at 10.1186/s12951-021-01205-8.

## Background

Hepatocellular carcinoma (HCC) is the second leading cause of cancer-related deaths worldwide [1]. The etiology of HCC including hepatitis B virus infection, hepatitis C virus infection, alcoholic liver disease, metabolic syndrome and aflatoxin exposure usually causes injury and chronic inflammation in liver [2]. One unique feature of HCC is its close association with chronic inflammation induced liver fibrosis. More than 80% of HCC develop in fibrotic or cirrhotic livers, suggesting an important role of liver fibrosis in the premalignant environment of the liver [3]. Besides radical treatment in early-stage HCC, there is still an unmet clinical need for the majority of unresectable HCC. Unfortunately, most of them do not respond well to either multi-kinase inhibitors or immune-checkpoint blocking antibodies, which are effective standard cares for other cancers [4]. This failure is primarily due to the fibrotic microenvironment and the related immunosuppressive factors in HCC [5]. Thus, stromal microenvironment regression together with immunosuppressive microenvironment remodeling will pose a potential strategy to HCC therapy.

Liver sinusoidal endothelial cells (LSECs) constitute a unique vascular bed in the liver, which receives the blood from both hepatic artery and portal veins into hepatic parenchyma [6]. LSECs are the first liver cell type affected after liver injury, and play a critical role in the cellular crosstalk that regulates progressive chronic liver disease, which leads to fibrosis and carcinogenesis [7]. Under physiological condition, differentiated LSECs function as gatekeeper to prevent hepatic stellate cells (HSCs) from activation. While the LSEC differentiation usually lost prior to fibrosis during pathological process, so-called capillarization, which amplifies liver damage by orchestrating the response of the liver microenvironment, permits and promotes HSCs activation, leading to liver fibrosis and carcinogenesis [8]. Reversal of capillarized LSECs to differentiated LSECs promotes reversion of activated HSCs (aHSCs) to quiescence and induces senescence and apoptosis of aHSCs, which is critical for the treatment of both fibrosis and HCC. In addition, LSECs influence the composition of hepatic immune populations by distinct expression of adhesion molecules and chemokines in diseased liver, which indicates the crucial role of LSECs in immune-regulation [9]. The characteristic of LSECs in contributing to HSC deactivation and immune response initiation makes them a potential therapeutic target for HCC treatment.

Statins, the 3-hydroxy-3-methylglutaryl CoA (HMG-CoA) reductase inhibitors which decrease cholesterol synthesis, are usually used to treat dyslipidemia and cardiovascular disease. Recently, there is accumulating evidence that statins have beneficial effects in liver fibrosis and cirrhosis [10, 11]. Results of cohort studies have consistently found that liver cirrhotic patients who received statin treatment to reduce cholesterol had a lower risk of decompensation and death compared with patients who did not receive statins [12–14]. Emerging clinical observational studies have also shown that statin use was associated with a reduced risk of HCC development and related mortality [15–17]. Simvastatin, as the most effective statin protecting the hepatic endothelium, was revealed to activate the transcription factor Kruppel-like factor (KLF2)-nitric oxide (NO) pathway to reverse LSEC capillarization and mediate hepatic endothelial protection [18, 19]. Mannose receptor is the typical scavenger receptors with high expression on LSECs, and mannan exclusively acts as a ligand to target the mannose receptor, being ability to facilitate simvastatin into LSECs [25]. Thus, the specific manipulation of this important switch in selected LSECs might be more effective than oral administration of statin nowadays as a strategy to treat HCC.

Here, we demonstrate that LSEC capillarization is associated with fibrotic HCC progression and poor survival. Simvastatin converts HSCs from activation to quiescence by stimulating KLF2-NO signaling in LSECs and up-regulates the expression of Chemokine (C-X-C motif) ligand 16 (CXCL16) on LSECs. In fibrotic HCC mouse model, targeted transfer of simvastatin into LSECs by nanotechnology not only mitigates LSEC capillarization to improve the stromal microenvironment, but also recruits natural killer T (NKT) cells to suppress tumor progression. Combined with anti-PD-L1 antibody, simvastatin nanoparticles (NPs) evoke an improved therapeutic effect at hemi-splenic inoculated advanced-stage HCC. These findings unravel an immune-based therapeutic mechanism of simvastatin and provide a novel therapeutic strategy based on tumor microenvironment remodeling against HCC.

## Materials and methods

### Human specimens

Medical history from 26 non-fibrotic HCC patients and 38 fibrotic HCC patients were collected for survival analysis. Immunohistochemistry were performed in a subset of patients with fibrotic HCC or non-fibrotic HCC. All 64 patients with HCC were obtained from Shuguang Hospital affiliated to Shanghai University of Traditional Chinese Medicine (SUTCM) and approved by SHUTCM Clinical Research Ethics Committee.

### Cell culture

Mouse hepatoma cell line Hepa1-6, human hepatocellular carcinoma cell line Huh7, LSEC cell line SK-Hep1 and HSC cell line LX2 were maintained in high glucose Dulbecco’s modified Eagle Medium (DMEM, Gibco) supplemented with 10% bovine calf serum (BCS) (Hyclone), 1% penicillin and streptomycin (Hyclone). Two μg/mL puromycin was used additionally for Hepa1-6-luciferase (luc) cells. All cells were grown at 37 °C in a humidified incubator with 5% CO_2_.

### Reagents

Simvastatin with over 99% purity (Sigma-Aldrich MO, USA) were used for the study. Mannan and L-NAME were purchased from Sigma-Aldrich. DiD was purchased from ThermoFisher Scientific. Poly(lactic-co-glycolic acid (PLGA)-Polyethylene glycol (PEG)-NH2 was purchased from NANOSOFT POLYMERS. Other synthetic materials as previously reported were obtained from Sigma-Aldrich MO, USA [20].

### Antibodies

In vivo anti-mouse IgG, CD1d, CD8 and CD4 mAb were purchased from BioXcell (West Lebanon, NH). Primary and secondary antibodies used for immunoblotting, immunostaining and flow cytometry are listed in Additional file 1: Table S1.

### Murine HCC models establishment

Four-week-old male C57BL/6 mice were purchased from Jackson Laboratory. All animal regulations and procedures were accepted by the Institutional Animal Care and Use Committee of the University of North Carolina at Chapel Hill. For fibrotic HCC model, 4-week-old male immune-competent C57BL/6 mice received intraperitoneal injection of CCl_4_ (1 μL/g, diluted in 100 μL olive oil) twice a week for 4 weeks. Age-matched mice administered with olive oil served as control. Intrahepatic injection of 5 × 10^6^ Hepa1-6-luc cells in 50 μL PBS was performed at the end of the fourth week. Mice were sacrificed at 3 weeks post-tumor cell inoculation or at humane endpoint. The tumors were isolated from the liver and measured with caliper. The volume was calculated with the formula of length × width × width/2, and the average value of each group was equal to the sum of total volumes divided by the number of mice. The tumor growth indicated by luciferase intensity was monitored using IVIS (Perkin Elmer, CA) imaging every 2 days. Tumor and matched non-tumor liver tissues were collected for section analysis. For advanced-stage HCC model, hemi-splenic injection of Hepa1-6 cells was performed after 4-week CCl_4_ exposure. To be specific, an incision located below the left rib cage was made to exteriorize the spleen. The spleen was tied and cut into two parts, each containing an intact vascular pedicle for each half of the spleen. The distal section of the spleen was inoculated with 1 × 10^6^ Hepa1-6 cells in 150 μL PBS. The half spleen containing inoculated cells were resected 5 min after inoculation allowing the cancer cells to enter the portal vein. The other half of the spleen was returned to the cavity to keep the immune system competent.

### Synthesis and characterization of the drug-loaded mannan modified NPs

The PLGA copolymers were obtained by a previously reported method [20, 21]. The PLGA-PEG and PLGA-PEG-MAN were first synthesized. Mannan attachment to the particle surface was achieved through covalent attachment by conjugation chemistry of the COOH-terminus of PLGA and mannan [22].

Two mg simvastatin was dissolved in 200 µL DMSO, and then mixed with 2 mg PLGA-PEG-MAN and 6 mg PLGA-PEG in the drug solution. The drug-polymer mixture was added dropwise to 10 mL deionized water while stirring, and stirred for 4 h. Nanoparticles were purified by ultrafiltration using 10 k cutoff columns at 4000×*g*.

The particle size and zeta potential of nanoparticle were measured by Malvern Nano-ZS (Malvern Instruments, UK). The morphology was analyzed by transmission electron microscopy (TEM). Five μL particles were added on 400-mesh carbon-filmed copper grids (Agar Scientific) for 2 min and stained with 2% (w/w) uranyl acetate before imaging using TEM (JEOL JEM1230).

The drug encapsulation efficiency (EE) was calculated by (Entrapped Drug/Drug Added)*100%. Loading capacity (LC) was calculated by (Entrapped Drug/Nanoparticle Weight)*100%.

### In vivo treatment studies

For intra-hepatic inoculation HCC model, the mice with liver tumors were treated 3 days after the tumor cell inoculation. Five groups of mice were separately given PBS, blank PLGA NPs, simvastatin PLGA NPs (20 mg/kg), simvastatin PLGA-MAN NPs (20 mg/kg) by tail vein injection and simvastatin free drug oral gavage (40 mg/kg) every other day for totally 5 times. The anti-tumor efficacy was regularly assessed using IVIS (Perkin Elmer, CA) imaging every 2 days and survival was recorded. Some mice were sacrificed 3 days after treatment, the tumors and matched non-tumor liver tissues were collected and prepared for section study and immune effect evaluation. For immune cell depletion study, after Hepa1-6-Luc intrahepatic inoculation, PBS, IgG, anti-CD1d, anti-CD4 or anti-CD8 antibody (100 µg) was intraperitoneally injected to mice every other day for totally 5 injections 1 day before NPs treatment. For hemi-splenic inoculation HCC model, intervention was applied 5 days after tumor cell inoculation. Four groups of mice were given separately by PBS, simvastatin PLGA-MAN NPs (20 mg/kg), anti-PD-L1 antibody (100 µg) or combination. NPs tail vein injection and anti-PD-L1 antibody intraperitoneal injection were performed every other day for a total 5 times.

### Mouse liver sinusoidal endothelial cell isolation

Mouse LSECs were isolated as previously described [23]. In brief, livers were perfused through the portal vein and digested with a collagenase solution. After mincing the liver, cells were filtered and centrifuged at 50×*g* to remove hepatocytes. Non-parenchymal cells were then separated by differential centrifugation using a Percoll gradient. Kupffer cells were eliminated by plastic pre-culture for 30 min. LSEC were collected for RNA extraction.

### Gene expression profiling

SK-Hep1 cells in triplicate were treated with simvastatin (20 μM) and control for 24 h. Total RNA was extracted using Trizol reagent (Invitrogen, Carlsbad, CA) according to the manufacturer’s protocol. RNA quality was assessed on an Agilent 2100 Bioanalyzer (Agilent Technologies, Palo Alto, CA) and checked using RNase free agarose gel electrophoresis. mRNAs were isolated and fragmented to about 200 base pairs length and reverse transcribed into cDNA using the QuantiTect Reverse Transcription kit (Qiagen). The cDNA fragments were purified with QiaQuick PCR extraction kit (Qiagen, Venlo, Netherland), end repaired, poly(A) added, and ligated to Illumina sequencing adapters. The cDNA library products were size selected by agarose gel electrophoresis, PCR amplified and sequenced using Illumina HiSeqTM4000 platform (Illumina, San Diego, CA). Transcript-level expression analysis of sequencing data was performed using HISAT and StringTie software (http://ccb.jhu.edu/software,shtml) [24]. Differential transcripts of chemokines with stringent cutoff coefficient of less than 0.05 were obtained to align to GO database (http://www.geneontology.org) for protein functional annotation corresponding to immune population.

### MTT assay

Cell viabilities were assessed by MTT assay. 10^4 cells were seeded in 96-well plates per well overnight and subjected to different treatments. Five mg/mL MTT (Alfa Aesar) reagent was added for 4 h at 37 °C, and then the supernatant was discarded. The formazan was resuspended in 100 μL of DMSO and absorbance was examined by a spectrometer (Hidex Chameleon).

### Histology

Liver tissues with tumor or major organs including hearts, livers, spleens, lungs, and kidneys were collected and were fixed in 4% paraformaldehyde (PFA). Fixed samples were paraffin-embedded, sectioned, and stained with hematoxylin and eosin (H&E) or Masson’s trichrome at UNC histology facility. PFA-fixed samples were embedded with optimum cutting temperature compound and sectioned at 8 μm thickness. For immunohistochemistry (IHC), sections were incubated with primary antibodies at 4 °C overnight, washed, and incubated with horseradish peroxidase-conjugated secondary antibodies for 2 h at room temperature. Digital images were taken using brightfield light microscope (Olympus BX61). Immunofluorescence (IF) was performed using fluorescent antibodies and counterstained with Prolong Diamond Antifade Mountant with DAPI (ThermoFisher Scientific). Antibodies are listed in Additional file 1: Table S1. Apoptotic cells were stained with a terminal deoxynucleotidyltransferase-mediated dUTP nick end labeling (TUNEL) kit (Promega, Madison, WI). Images were taken using laser-scanning confocal fluorescence microscope (Zeiss LSM 710). Liver samples fixed with 4% PFA by perfusion through portal vein were sectioned using vibratome at UNC microscopy services laboratory and prepared for scanning electron microscopy (Zeiss Supra 25 FESEM). Five random microscopic fields were selected and quantified by ImageJ software. Porosity was measured as percentage of LSEC surface occupied by fenestrae in SEM in liver tissue. Fenestration frequency was calculated with total number of fenestrations divided by total area of LSEC surface.

### Flow cytometric analysis

Single-cell suspensions from tumor tissue were harvested in MACs buffer (1 × PBS + 2 mM EDTA + 0.5% BSA, filter sterile), then subjected to conjugated staining with fluorescence. At least 10,000 live cells were subjected to flow cytometric analysis on a flow cytometer (Becton Dickinson LSR II). Experimental data were analyzed using FlowJo software. The antibodies used are listed in Additional file 1: Table S1.

### Immunoblotting

Cells were lysed in RIPA lysis buffer with protease inhibitors. Total lysates were quantified by a BCA Protein Assay Kit (Biorad, CA). Thirty μg protein samples were used for immunoblotting analysis. After incubating with appropriate primary and secondary antibodies, the immunoreactions were visualized with Western HRP substrate (Thermo, Rockford, IL). The antibodies used are listed in Additional file 1: Table S1.

### Quantitative real-time polymerase chain reaction (RT-PCR) assay

Total RNA was extracted from cells or the whole tumor using an RNeasy microarray mini kit (Qiagen, Hilden, Germany) and was reverse-transcribed to cDNA with an iScript cDNA synthesis kit (Bio-Rad, Hercules, CA). Quantitative PCR was performed in a 7500 RT-PCR system. The PCR primers are listed in Additional file 1: Tables S2 and S3.

### Nitric oxide assay

Nitric oxide (NO) amount was assessed using Nitric Oxide Colorimetric Assay Kit (BioVision, Milpitas, CA) in accordance to the manufacturer’s manual. Briefly, SK-Hep1 cells were treated with different concentrations of simvastatin (0, 5, 20 μM) for 24 h and the supernatants were collected. Equal aliquot of samples in every group were mixed with Nitrate Reductase Buffer and incubated at room temperature for 1 h. The mixture was then added Griess Reagent and color was developed in 10 min. The absorbance was measured at 540 nm and NO concentration was calculated referring to a standard curve. The experiments were done in triplicate.

### Biodistribution

0.05% (wt) of lipophilic carbocyanine DiD (ThermoFisher) was used to formulate the DiD-labeled PLGA-PEG or PLGA-PEG-MAN NP. In mice bearing fibrotic tumor, 24 h following tail vein injection of DiD-labeled NP, major organs and liver with tumors were collected and analyzed using IVIS (Perkin Elmer, CA) with the excitation wavelength at 640 nm and the emission wavelength at 670 nm.

### Blood chemistry analysis

Seven days after 5 doses of nanoparticle injections, whole blood was obtained from the normal mice. Blood routine including RBC, WBC, PLT, HGB, and HCT was tested using the whole blood. Aspartate aminotransferase (AST), alanine aminotransferase (ALT), urea nitrogen (BUN), and creatinine (CRE) in serum were analyzed as indicators of hepatic and renal function using the serum.

### Statistical analysis

GraphPad Prism 8 (GraphPad Software, La Jolla, California, USA) was used for statistical analysis. The independent Student t-test was used to compare data between the two groups. A one-way analysis of variance test with Bonferroni correction was used to compare data in multiple groups of mice. Pearson correlation coefficient was calculated to show the correlation of two parameters. Two-way ANOVA with a Bonferroni post-hoc test was used to estimate the difference in multiple groups of mice when considering two factors of time and treatment. Kaplan–Meier survival analysis was used to determine the overall survival rates and tumor incidence-free rates, the differences were compared by the log-rank Mantel-Cox test. Data averages from each group are presented as mean ± SD; *p < 0.05, **p < 0. 01, ***p < 0. 001.

## Results

### Liver fibrosis-associated LSEC capillarization contributes to aggressive HCC development

**Fig. 1:**
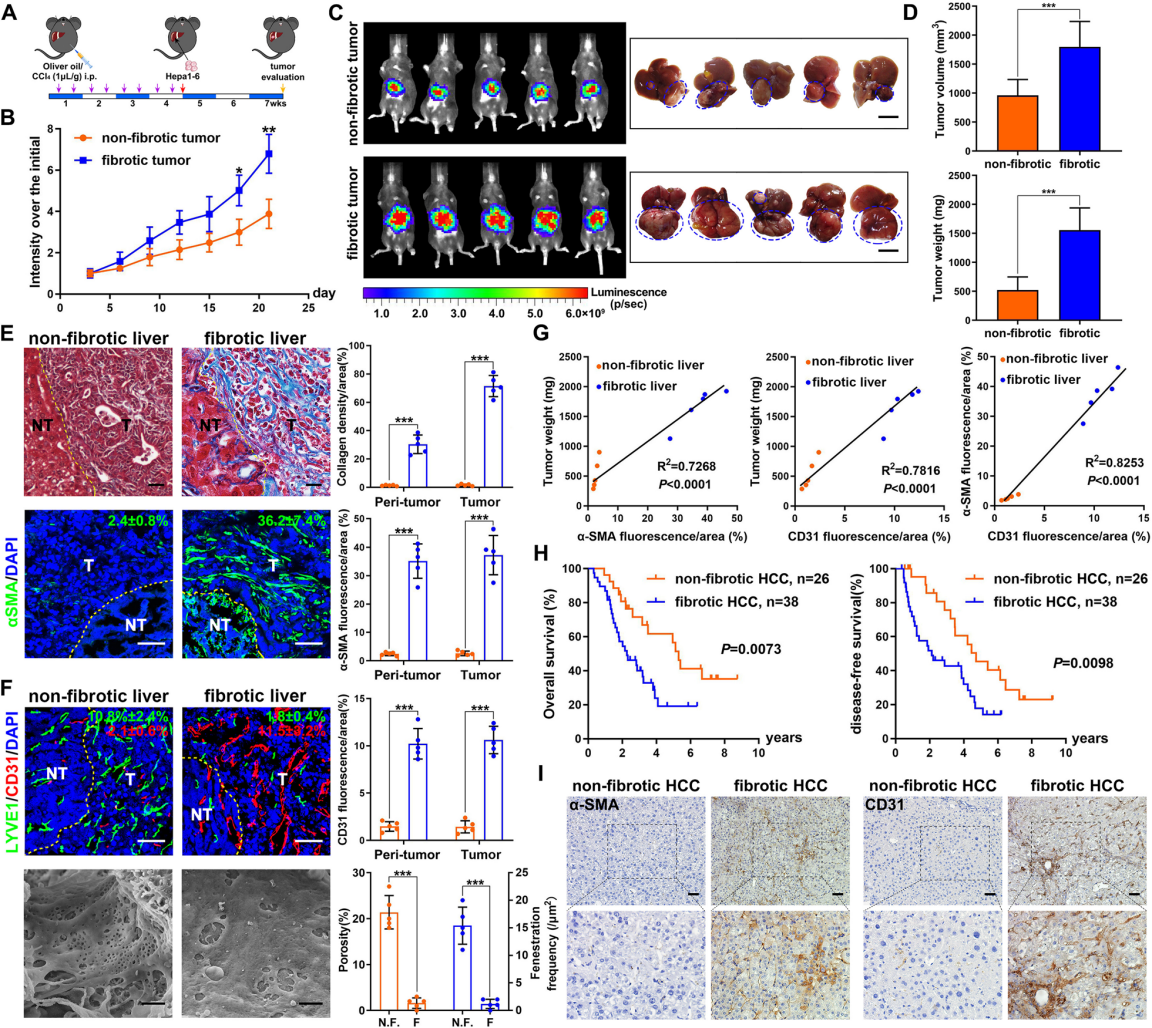
Liver fibrosis-associated LSEC capillarization contributes to aggressive HCC development. **A** Schematic diagram of liver fibrosis-associated HCC mouse model. **B** Tumor growth curve and **C** Bioluminescence imaging of tumor and representative liver tumor morphology at the endpoint. **D** Statistical analysis of tumor volume and weight. **E** Representative pictures of Masson trichrome staining, α-SMA immunofluorescence in mice non-fibrotic and fibrotic liver tissue. Scale bar stands for 50 μm. Yellow dotted line indicates the border between non-tumor and tumor tissue. The quantification of collagen density, α-SMA expression were shown. **F** LYVE1 and CD31 co-immunofluorescence in mice non-fibrotic and fibrotic liver tissue, scale bar stands for 50 μm. Scanning electron micrographs illustrating the morphological response of LSEC. Scale bar stands for 1 μm. The quantification of LYVE1 and CD31 expression were shown, as well as the porosity and fenestration on LSEC. **G** Percentage of α-SMA, CD31 fluorescence and tumor weight are pairwise positively correlated. **H** Kaplan–Meier overall survival and disease-free survival curves of HCC patients with non-fibrotic tumor (n = 26) or fibrotic tumor (n = 38). **I** Expressions of α-SMA and CD31 in non-fibrotic HCC and fibrotic HCC tissues as determined by immunohistochemistry. Scale bar stands for 50 μm.*p < 0.05, **p < 0.01, ***p < 0.001

To observe the role of fibrotic microenvironment during HCC development, Hepa1-6 orthotopic HCC model was established using C57BL/6 mice pretreated with CCl_4_ injection which induces chronic inflammation and fibrosis (Fig. 1A). Interestingly, tumor growth accelerated more rapidly in fibrotic liver than the normal liver, as shown by the tumor growth curve (Fig. 1B) and bioluminescence imaging of tumors, as well as liver tumor morphology at the endpoint (Fig. 1C). Tumor weight and volume are confirmed as well (Fig. 1D).

To further investigate the fibrotic microenvironment responsible for HCC development, we first evaluated the expression of α-smooth muscle actin (αSMA) and collagen which are key markers of aHSCs and fibrosis. Both markers were highly expressed in peri-tumor and tumor tissues in CCl_4_-treated group as expected (Fig. 1E). Since LSECs play critical role during fibrosis, lymphatic vessel endothelial hyaluronan receptor 1 (LYVE1) and CD31 which are the markers of differentiated and capillarization LSECs, respectively, were assessed. Interestingly, high LYVE1 expression was observed in both peri-tumor and tumor tissues of non-fibrotic liver, while CD31 was over-expressed in fibrotic liver (Fig. 1F). The porosity and fenestration on LSECs were much reduced in fibrotic liver compared to the non-fibrotic liver (Fig. 1F). We further found that αSMA, CD31 expression level and tumor mass were significantly pairwise positively correlated (Fig. 1G), while LYVE1 expression was negatively correlated with tumor mass. Thus, our results showed LSEC capillarization may contribute to aggressive HCC development.

We next investigated the clinical relevance of LSEC capillarization in HCC patients with or without fibrosis/cirrhosis. The Kaplan–Meier analysis revealed that HCC patients with fibrosis were significantly correlated with shorter overall and disease-free survival rates (Fig. 1H). Consistent with the mouse model, the fibrotic HCC tissue exhibited high expression level of αSMA and CD31 (Fig. 1I). Thus, poor survival in fibrotic HCC patients is related to LSEC capillarization.

### Simvastatin deactivates HSC via LSEC and up-regulates CXCL16 expression on LSEC

**Fig. 2:**
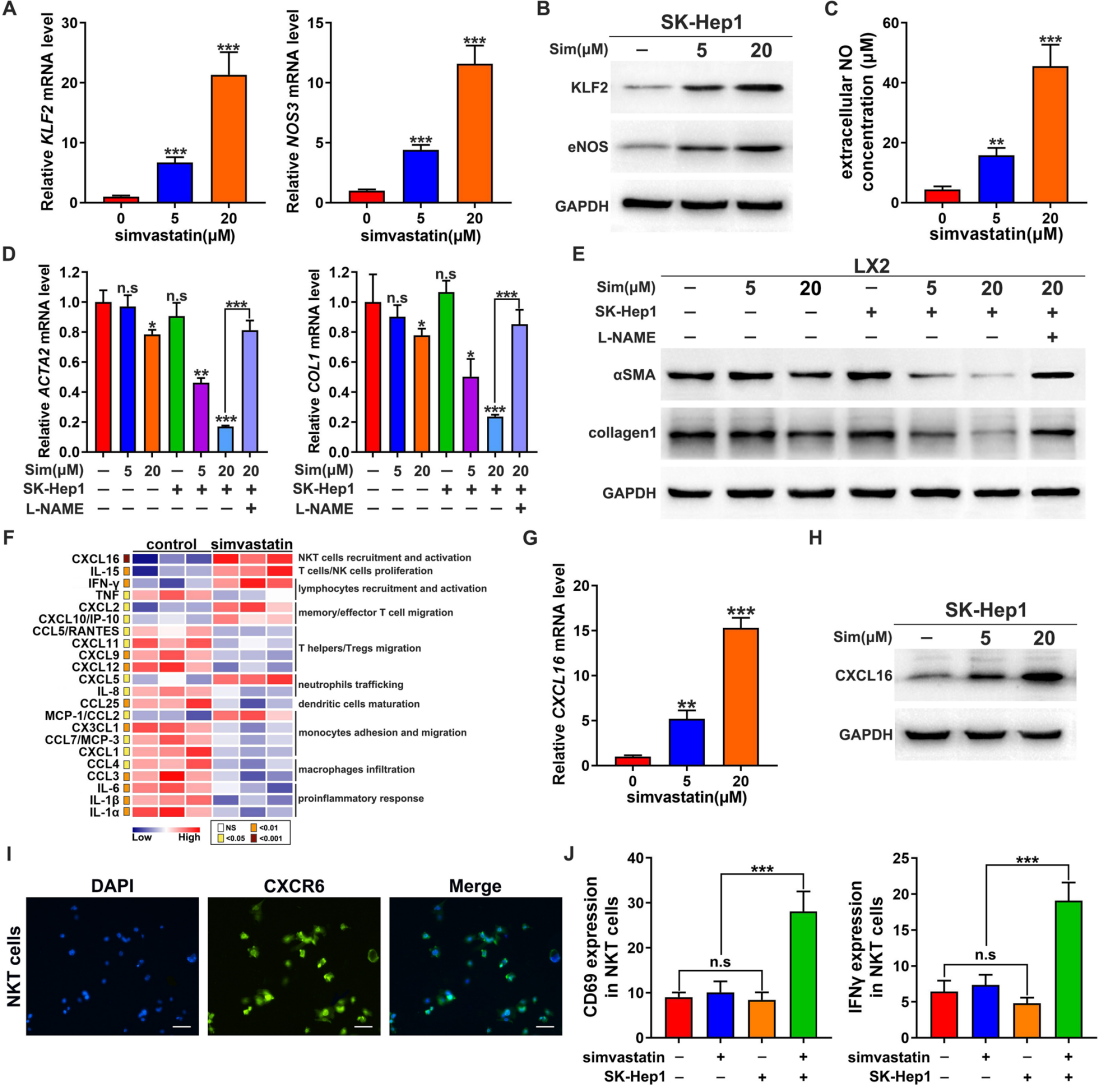
Simvastatin deactivates HSC via LSEC and recruits NKT cells. **A** Quantitative RT-PCR analysis and **B** immunoblotting of KLF2 and eNOS in SK-Hep1 LSEC cells treated with the indicated concentrations of simvastatin for 24 h (n = 3). GAPDH acts as the loading control. **C** Extracellular concentration of NO upon different doses of simvastatin treatment (n = 3). **D** Quantitative RT-PCR analysis and, **E** immunoblotting of *ACTA2* and *COL1* in LPS-activated LX2 treated with the supernatant of SK-Hep1 upon different doses of simvastatin treatment with or without L-NAME (n = 3). GAPDH acts as the loading control. **F** Gene expression profiles of chemokines analyzed with RNA-seq data which derived from SK-Hep1 cells treated with simvastatin or control. Simvastatin-responsive chemokine genes were grouped in association with clusters of various immune cell-regulating functions. The *p* value of each comparison was indicated with colors. **G** Quantitative RT-PCR analysis and **H** immunoblotting of *CXCL16* in SK-Hep1 cells treated with the indicated concentrations of simvastatin for 24 h (n = 3). GAPDH acts as the loading control. **I** CXCR6 expression was detected in NKT cells. **J** The expression of CD69 and IFN-γ was detected in NKT cells treated with control, simvastatin, the co-culture with SK-Hep1 cells, and the combination, respectively (n = 3). *p < 0.05, **p < 0.01, ***p < 0.001, n.s. = not significant

Since simvastatin exerts beneficial effects in fibrosis and HCC, we first examined the effect of simvastatin on three human cell lines which are SK-Hep1 LSEC cell line, LX2 HSC cell line and Huh7 HCC cell line. We found simvastatin almost had no cytotoxicity to all three cell lines (Additional file 1: Fig. S1). However, simvastatin increased both mRNA and protein expression levels of KLF2 and endothelial nitric oxide synthase (eNOS) in SK-Hep1 cell line in a dose dependent manner (Fig. 2A, B and Additional file 1: Fig. S2A). The NO excretion was up-regulated according to the activation of KLF2-eNOS signaling induced by simvastatin (Fig. 2C). When lipopolysaccharide (LPS)-activated LX2 treated with simvastatin directly, no change of the activation marker αSMA and collagen was observed. Since simvastatin increased NO excretion in SK-Hep1, we collected the conditioned medium of simvastatin treated SK-Hep1 cells and added to cultured LX2 cells. The mRNA and protein expression levels of αSMA and collagen I were both down-regulated dose-dependently (Fig. 2D, E and Additional file 1: Fig. S2B). Further, an NOS inhibitor L-NAME was used to verify the NO induced-HSC deactivation. Indeed, LX2 activation recovered after L-NAME intervention (Fig. 2D, E and Additional file 1: Fig. S2B). Thus, simvastatin seemed to work on SK-Hep1 cells which then secreted NO, or an NO-related factor, to deactivate LX2 cells.

In addition, since LSECs play critical roles in the composition of hepatic immune populations by mediating the recruitment of leukocyte subsets through the expression of distinct chemokines, we performed RNA-sequencing (RNA-seq) in simvastatin treated SK-Hep1 cells, or not, to examine whether simvastatin affect the immune-regulated genes in LSEC. Gene expression profiles analysis of RNA-seq data suggested that a set of chemokines, which are classified in terms of the function on the regulation of the variety of leukocytes, were changed in SK-Hep1 cells treated with simvastatin compared with the untreated control cells. The investigation coupled with functional categorization of changed chemokine genes revealed that CXCL16 was identified as the significant regulator upon simvastatin treatment (Fig. 2F). Further quantitative PCR and western blot detection confirmed that transcript level and protein expression of CXCL16 greatly increased in SK-Hep1 treated with simvastatin in a dose-dependent manner (Fig. 2G, H and Additional file 1: Fig. S2C). NKT cells were characterized with high expression of CXCR6 (Fig. 2I), and CXCL16 as the ligand for CXCR6 exerts recruitment function on NKT cells. Further study indicated that simvastatin alone could not influence the activity of NKT cells, while simvastatin could upregulate the expression of CD69 and IFN-γ in NKT cells in the co-culture with SK-Hep1, suggesting simvastatin evoked the antitumor NKT immune response through the mediation of LSEC (Fig. 2J). Thus, simvastatin can not only deactivate HSCs via KLF2-NO signaling in LSECs, but also induce CXCL16 overexpression in LSECs which recruits and activates NKT cells to the liver.

### Design of simvastatin LSEC-targeted delivery NP platform

**Fig. 3:**
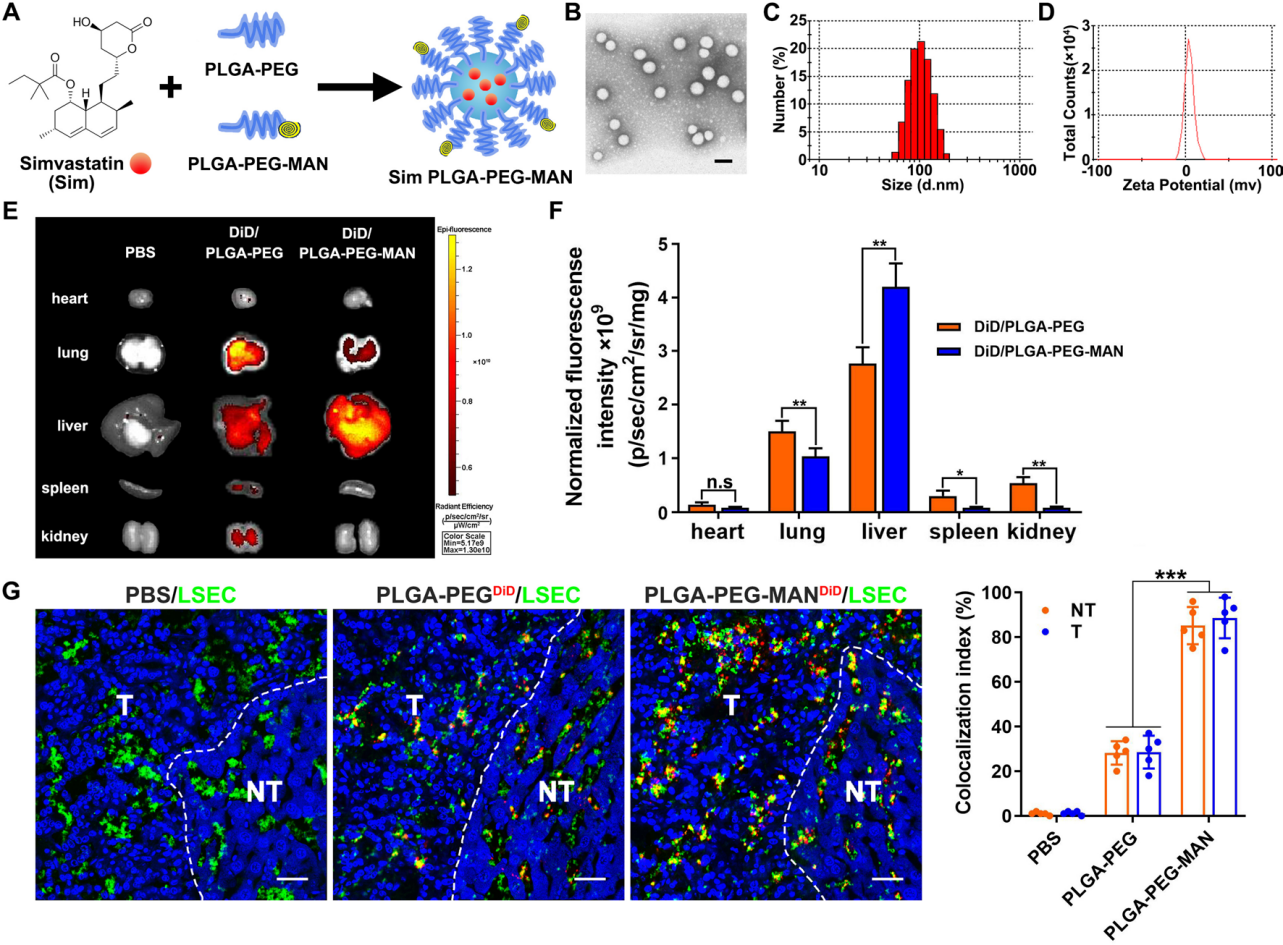
Simvastatin LSEC-targeted delivery NP platform was designed. **A** Efficient co-encapsulation of simvastatin into PLGA NP using a solvent displacement technique. **B** TEM images of PLGA NPs loaded with simvastatin cores. **C** Particle size of the PLGA NP. **D** Zeta Potential of the PLGA NP. **E** Images and **F** quantification of the DiD-loaded PLGA (DiD/PLGA-PEG) and mannan conjugated PLGA(DiD/PLGA-PEG-MAN) in liver with tumor and other major organs at 24 h after injection in mice bearing Hepa1-6 liver tumor. **G** Fluorescence imaging detecting DiD in PLGA-PEG or mannan-coated PLGA-PEG and LSEC marked with CD31, scale bar stands for 50 μm. *p < 0.05, **p < 0.01, ***p < 0.001, n.s. = not significant

Simvastatin is poor soluble in aqueous solution. The PLGA polymer approved by FDA for drug delivery was selected for encapsulating hydrophobic simvastatin due to the characteristic of both biodegradability and biocompatibility. A unique characteristic of LSECs is their expression of high levels of scavenger receptors, which ensure the high endocytic capacity [7]. One of the most extensively studied scavenger receptors on LSECs is the mannose receptor [25]. Thus, mannan was selected as a ligand to target the mannose receptor on LSEC. The structure of synthetic PLGA-PEG-mannan was confirmed by NMR. The ^1^H NMR spectrum of PLGA-PEG-mannan showed that δ 4.639(H1), δ 3.51(H2), δ 5.20(H3)and δ 4.90(H4)are associated with CH of mannan, CH2 of PEG, CH of lactic acid and CH2 of glycolic acid, respectively (Additional file 1: Fig. S3), which indicated the success of synthesis. We utilized a solvent displacement process to load simvastatin (Fig. 3A). PLGA NPs containing simvastatin presented a micelle-like structure with a diameter of approximately 100 nm revealed by transmission electron microscopy (Fig. 3B). The particle size around 120 nm and zeta potential around 3 mV were confirmed by particle sizing system (Fig. 3C, D). The drug encapsulation efficacy of simvastatin was approximately 85%, loading capacity was 6.5%.

NPs with mannan target tend to accumulate much more in fibrotic liver with tumors, but less in other main organs than the non-targeted NPs at 24 h post tail vein injection, which was tracked using bioimaging (Fig. 3E, F). The uptake of NPs in fibrotic liver tissues was further examined by immunofluorescence staining. As expected, mannan-coated particles encapsulating DiD were captured by LSECs almost 4 times higher compared to non-coated ones both in tumor and non-tumor tissues (Fig. 3G).

### LSEC-targeted delivery of simvastatin inhibits tumor growth in mouse fibrotic HCC model

**Fig. 4:**
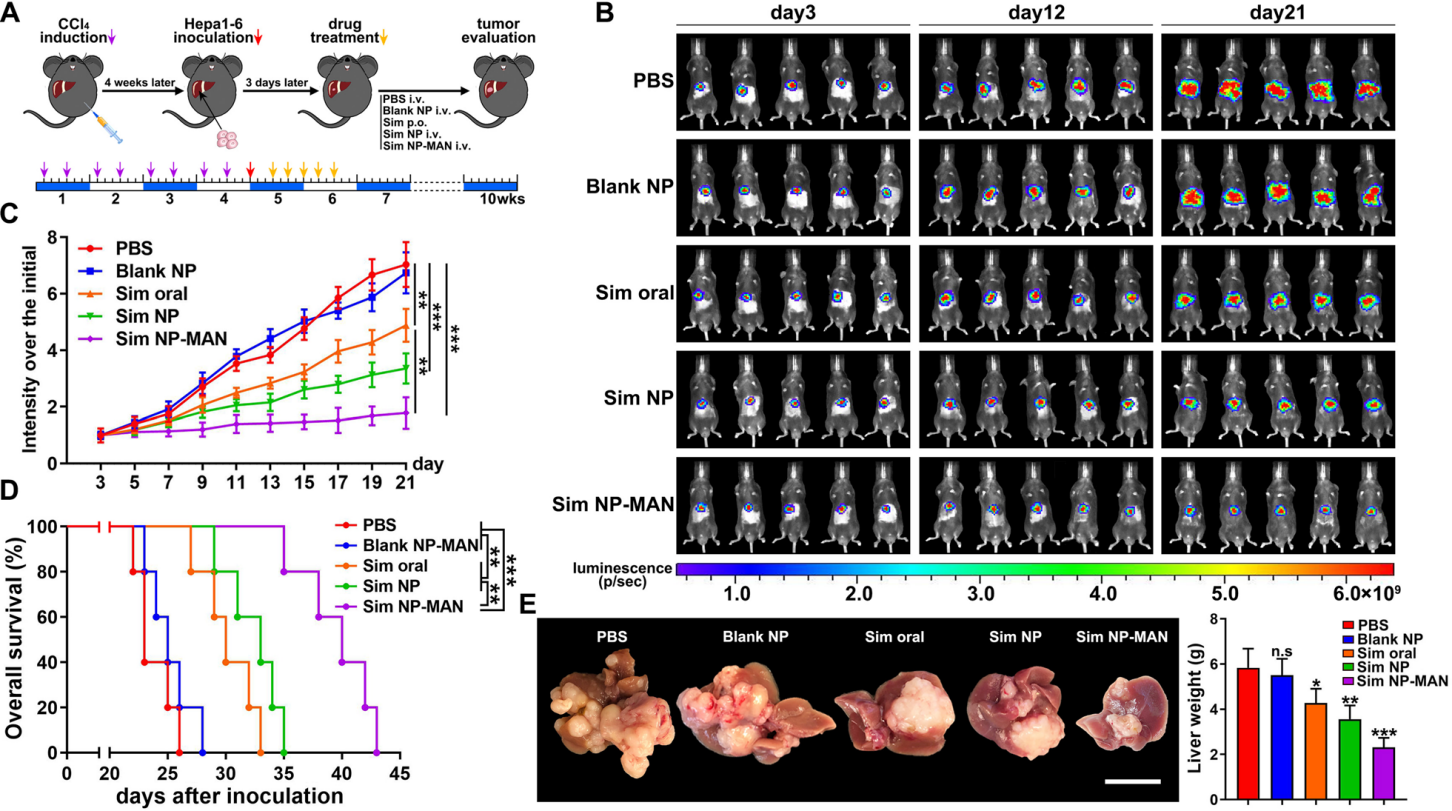
Simvastatin NPs suppress HCC development. **A** Tumor inoculation and treatment scheme. **B** Bioluminescence imaging of Hepa1-6 tumor bearing mice during tumor progression in various treatment groups. **C** Tumor growth curves and, **D** survival curves of Hepa1-6 tumor bearing mice in various treatment groups (n = 5). **E** Representative image of liver with Hepa1-6 tumor and quantification of liver weight at day 21 after inoculation (n = 3). Scale bar stands for 1 cm. *p < 0.05, **p < 0.01, ***p < 0.001, n.s. = not significant

To investigate the effect of targeted delivery of simvastatin in vivo, the Hepa1-6 orthotopic HCC model was established using C57BL/6 mice pretreated with CCl_4_. Three days after tumor cells inoculation, treatment was administrated every other day for continuous 5 times. Mice were treated by tail vein injection of PBS, blank NPs, simvastatin un-targeted NPs (Sim NP) (20 mg/kg), simvastatin LSEC-targeted NPs (Sim NP-MAN) (20 mg/kg) and simvastatin oral administration (40 mg/kg), respectively (Fig. 4A). Notably, Sim NP-MAN impressively mitigated rapid tumor growth compared to PBS or blank NP-treated group, as shown by the lower increased rate of tumorous luciferase intensity and tumor growth curves. Either simvastatin oral administration or Sim NPs injection exhibited partial inhibitory effect (Fig. 4B, C). Besides, a significant survival prolongation from 23 days in the control group to 43 days in the Sim NP-MAN treated group was also noticed (Fig. 4D). The livers with tumor were excised at day 21 for inspection, liver tumor load of the Sim NP-MAN treated group were significantly reduced than those of the other groups (Fig. 4E). Thus, LSEC-targeted delivery of simvastatin showed better anti-HCC effect than oral administration and un-targeted delivery.

### LSEC-targeted delivery of simvastatin remodels stromal environment and recruits NKT cells in fibrotic HCC

**Fig. 5:**
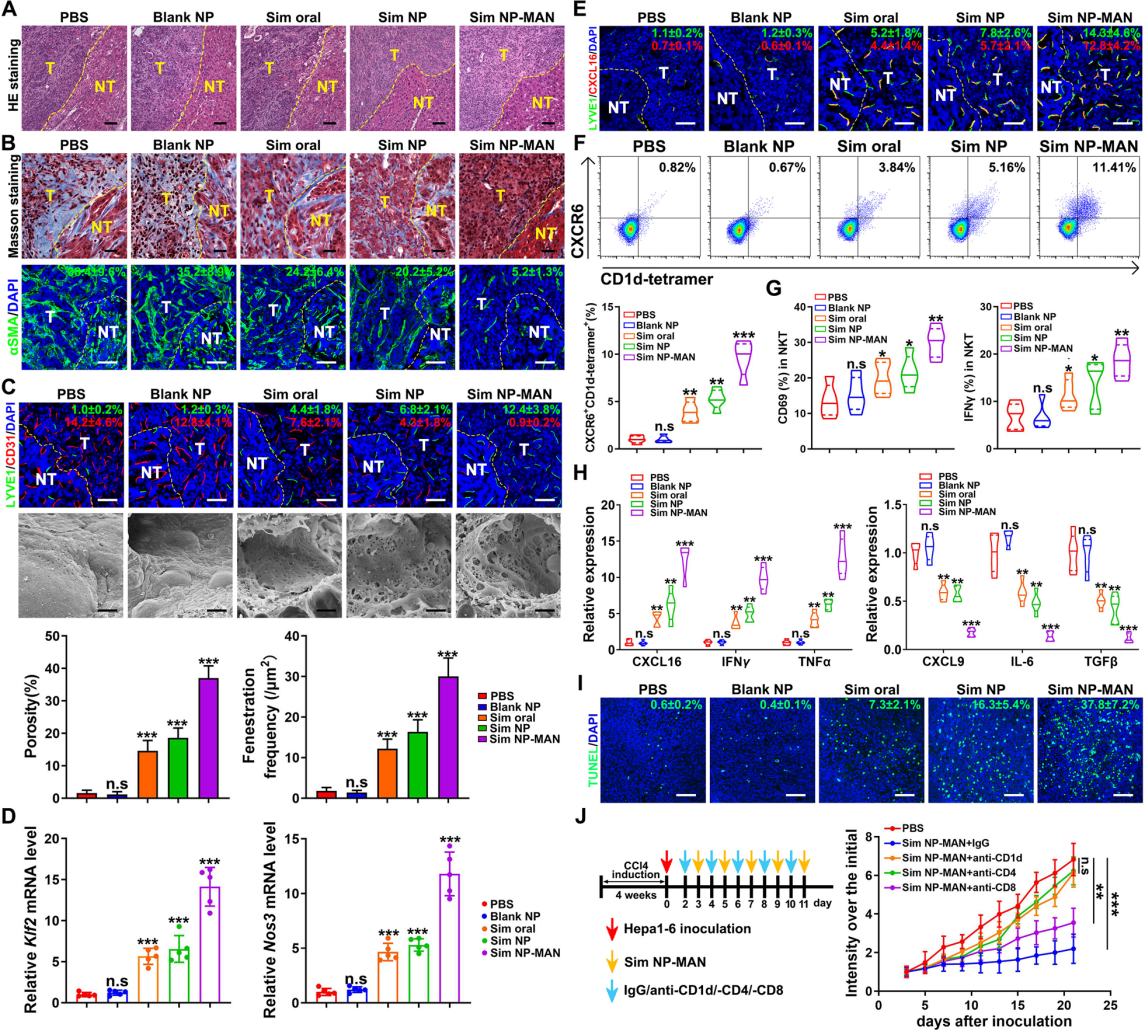
Simvastatin NPs remodel tumor microenvironment in fibrotic HCC. **A** HE staining of tissue features from HCC mice with various treatments. Yellow dotted line indicates the border between normal liver and tumor tissue. The left side is non-tumor liver tissue and right side is tumor tissue. Representative images are shown. Scale bar stands for 50 μm. **B** Masson trichrome (MT) staining shown together with αSMA fluorescence staining in liver tissues. Data are presented as mean ± SD. Scale bar in MT image stands for 20 μm, in IF image stands for 50 μm. **C** LYVE1 and CD31 expression by fluorescence staining shown together with LSEC feature using SEM in liver tissues. The quantification of porosity and fenestration of LSEC is shown (n = 5). Scale bar in IF image stands for 50 μm, in SEM image stands for 1 μm. **D** Quantitative RT-PCR analysis of *Klf2* and *Nos3* in LSECs extracted from liver tissues intervened with various treatments (n = 5). **E** The fluorescence staining of LYVE1 and CD31. Scale bar stands for 50 μm. **F** NKT cell frequency in liver tissues detected by flow cytometry (n = 5). **G** CD69^+^ and IFN-γ^+^ levels of hepatic NKT cells detected by flow cytometry (n = 5). **H** mRNA expression of chemokines and cytokines detected by real-time PCR in tumors in various treatment groups (n = 5). **I** The average number of apoptosis cells per high-power field (HPF) detected by TUNEL immune fluorescence staining. Scale bar stands for 50 μm. **J** Scheme of immune cell depletion study and tumor growth curves of Hepa1-6 tumor treated with simvastatin NP after anti-CD1d, CD4or CD8 intraperitoneal injection (n = 5). *p < 0.05, **p < 0.01, ***p < 0.001, n.s. = not significant

We next examined the effect of simvastatin delivered with mannan-targeted NPs to remodel stromal environment in the liver. HE staining revealed that the disorder structure of both tumor and non-tumor fibrosis tissue reversed in Sim NP-MAN treated group (Fig. 5A). The collagen deposition reduced significantly as shown in Masson-trichrome staining. Accordingly, the expression of αSMA was approximately sevenfold down-regulated compared to control group (Fig. 5B). The over-expressed LSEC capillarization marker CD31 were remarkably down-regulated, while differentiated marker LYVE1 up-regulated in Sim NP-MAN treated group. In addition, both porosity and fenestration frequency of LSECs increased significantly (Fig. 5C). Further, we found the mRNA expression levels of *Klf2* and *Nos3* in LSECs extracted from liver tissues were up-regulated significantly, which is consistent with the observation in vitro*,* indicating the *Klf2-NO* signaling were activated by targeted delivery of simvastatin (Fig. 5D). Since simvastatin-treated LSECs express CXCL16, we next examined the co-localization of CXCL16 and LYVE1 in liver tissue. As expected, CXCL16 expression increased together with LYVE1 after Sim NP-MAN treatment (Fig. 5E).

As CXCL16 is the ligand for CXCR6 which is expressed mostly on NKT cells, the cell frequencies of CXCR6^+^ NKT cell in tumor tissues of various groups were evaluated by flow cytometry. CXCR6^+^ NKT cell frequency increased from 0.82% in control group to 11.41% in Sim NP-MAN treated group (Fig. 5F). Both CD69 and IFN-γ which are the activation markers of NKT cells were up-regulated (Fig. 5G). We further examined the critical cytokines in tumor tissues of various groups. The increased expression of the immunostimulatory cytokines including CXCL16, IFN-γ, TNFα and the decrease of immunosuppressive cytokines including CXCL9, IL-6, TGFβ were noticed with Sim NP-MAN treatment (Fig. 5H). Also, the TUNEL assay revealed increased number of apoptotic cells in tumor tissues after Sim NP-MAN treatment (Fig. 5I). To address the immune mechanism, we further depleted NKT, CD4^+^ or CD8^+^ T cells in mice HCC model (Fig. 5J). The tumor restraining effect was abolished by treatment with anti-CD1d, anti-CD8 or anti-CD4 antibody, whereas it was not affected when an isotype-matched IgG control was used. CD1d and CD4 antibody showed the most obvious abolishment which indicates that NKT cells played a more critical role during the anti-tumor process than the CD8^+^ T-cells (Fig. 5J). Taken together, the result suggested that stromal environment re-modulation and NKT cell recruitment might be the main reason for the antitumor effect of Sim NP-MAN in fibrotic HCC.

### Simvastatin NPs show no obvious toxicity

To further evaluate the safety of simvastatin NPs for the development of both effective and translational therapy, biosafety-related toxicological serum and pathology analyses were performed. The pathology revealed by H&E staining showed no significant morphological damage among major organs including heart, lung, liver, spleen and kidney (Additional file 1: Fig. S4A). The body weight remained stable without severe weight loss between different groups during experiment (Additional file 1: Fig. S4B). The blood routine and hepatorenal function of mice in all groups remained in the normal range (Additional file 1: Fig. S4C, D). Thus, simvastatin NPs showed no obvious toxicity in the mouse model.

### Simvastatin NPs together with PD-L1 antibody achieve the synergistic effect in late-stage HCC

**Fig. 6:**
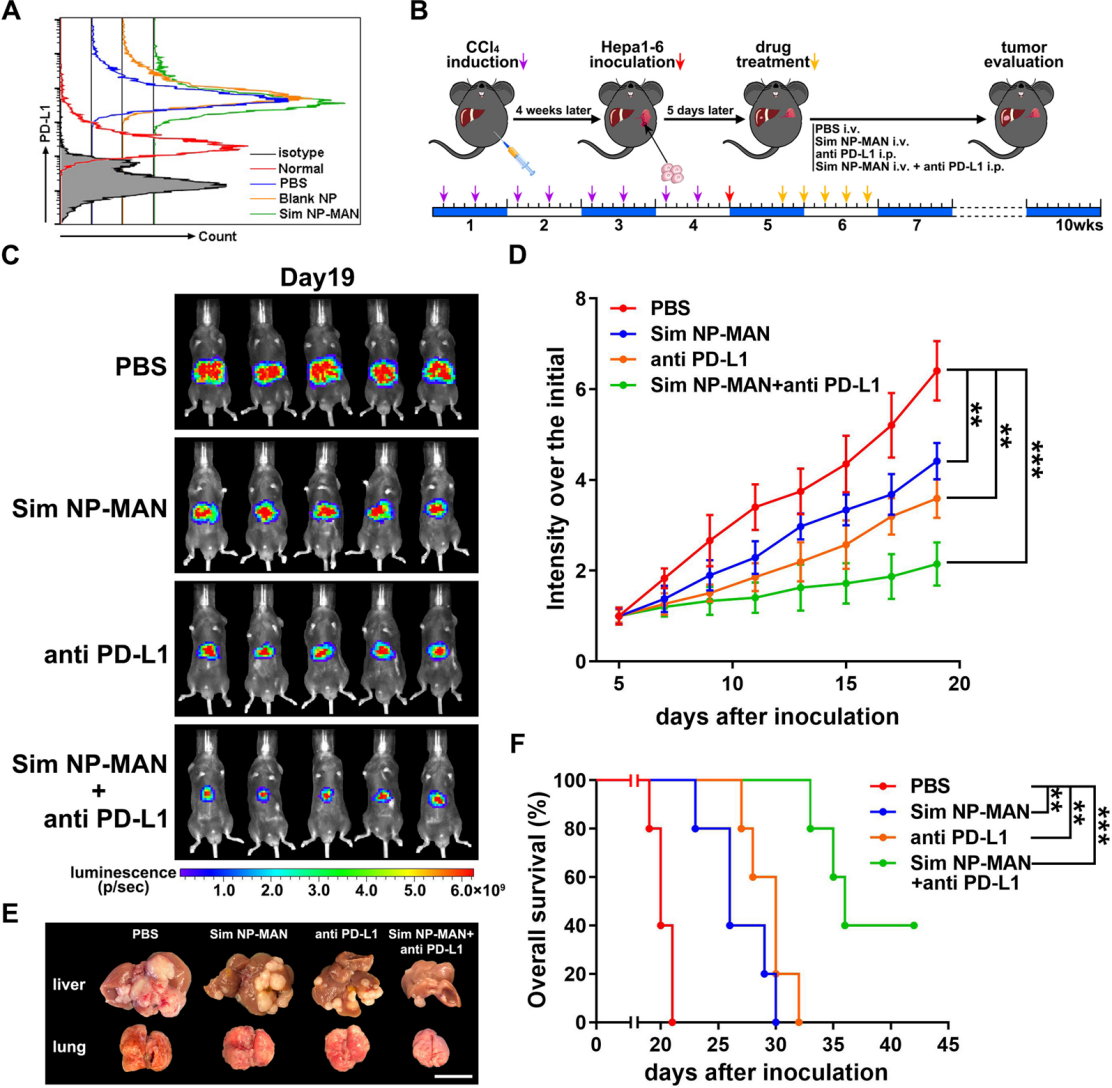
Combination of simvastatin NP and PD-L1 antibody shows synergistic efficacy in advanced HCC suppression. **A** PD-L1 level of HCC tissues from various treatments and normal liver were measured by flow cytometry. **B** Mice HCC model establishment and treatment scheme. **C** Bioluminescence imaging of Hepa1-6 tumor bearing mice in various treatment groups (n = 5). **D** Tumor growth curves. **E** Representative liver tumor morphology. Scale bar stands for 1 cm. **F** Survival curves (n = 5). **p < 0.01, ***p < 0.001, n.s. = not significant

Despite surveillance, HCC often presents in clinic at an advanced stage that only systemic therapy is feasible. Currently, most studies on the mechanisms of HCC immune evasion have focused on the programmed death receptor 1 (PD-1)/programmed death ligand 1 (PD-L1) pathway [26]. PD-1 is an immunoinhibitory receptor expressed in activated T and B cells, as well as NKT cells. PD-L1, the major ligand of PD-1, is expressed in various immune cells such as antigen-presenting cells, as well as in endothelial cells [27]. The PD-L1 expression was higher in Hepa1-6 tumor tissue compared to normal tissue, and simvastatin NPs showed no effect on PD-L1 expression (Fig. 6A). To explore an effective strategy for late-stage HCC treatment, the hemi-spleen inoculation of Hepa1-6 based on CCl_4_-induced fibrosis model was used to test the therapeutic effect of simvastatin NPs combined with PD-L1 antibody. In this aggressive HCC model, due to the dispersing of tumor cell to liver through splenic artery and portal vein, 20% of lung metastasis can be observed and mice usually die around day 20 if without treatment. Five days after inoculation, mice were treated with PBS, Sim NP-MAN (20 mg/kg, tail vein injection), PD-L1 antibody (100 μg, intraperitoneal injection), or both for totally 5 times every other day (Fig. 6B). Both Sim NP-MAN and PD-L1 antibody showed a slight effect in tumor suppression in HCC mice, whereas the combination of both treatments inhibited tumor progression significantly (Fig. 6C, D). Severe tumor development and lung metastasis could be observed at day 19 after Hepa1-6 inoculation, while much smaller tumor foci and no lung metastasis was found in group with combination treatment (Fig. 6E). The overall survival in the combination group extended almost 2 times of the control group (Fig. 6F). As expected, upon the same treatment of Sim NP-MAN, the prognosis of tumors in the advanced stage HCC model was much worse than intrahepatic inoculation HCC model. However, Sim NP-MAN combined with PD-L1 antibody still achieved a satisfactory therapeutic effect in the late-stage HCC model.

## Discussion

**Fig. 7:**
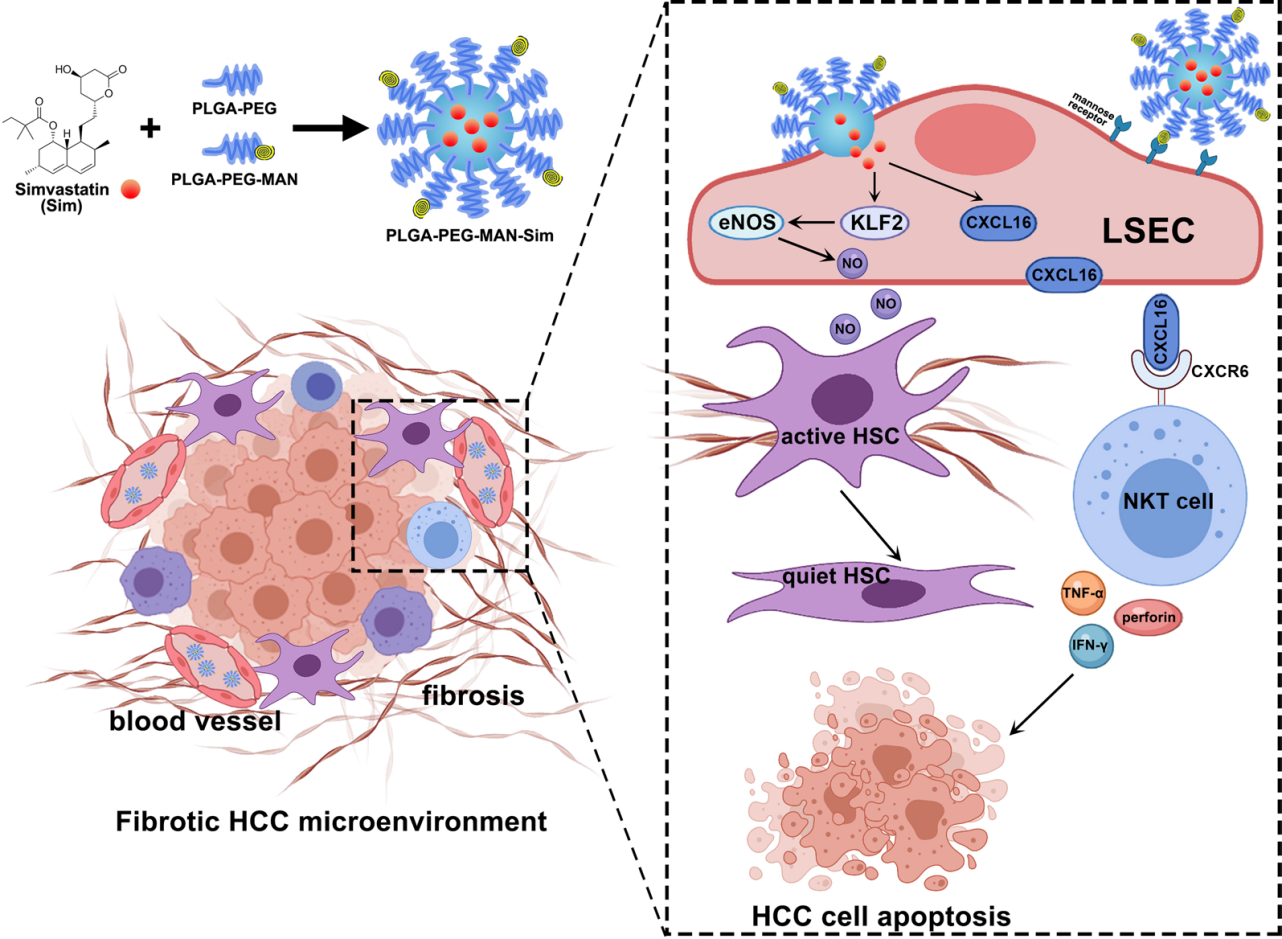
Schematic representation of the Nano delivery of simvastatin targeting LSECs to remodel HCC microenvironment

Our study indicated that LSEC capillarization accounts for tumor progression and poor survival in fibrotic HCC. Simvastatin functions as hepatic endothelium protector stimulates KLF2-NO signaling in LSECs to regress HSC activation. Simvastatin also up-regulates CXCL16 in LSECs which can recruit NKT cells. In order to exert the dual effect of simvastatin on LSEC, we targeted-delivered simvastatin to LSECs by a PLGA-PEG-MAN NP formulation. LSEC-targeted delivery of simvastatin not only alleviates LSEC capillarization to regress the stromal microenvironment, but also recruits NKT cells to remodel immunosuppressive microenvironment and inhibit tumor progression in murine HCC model (Fig. 7). In advanced-stage HCC, combination therapy of simvastatin NP and anti-PD-L1 antibody achieves an improved therapeutic effect.

LSECs are highly specialized endothelial cells in liver, which lies between blood cells on the sinusoidal side and hepatocytes and HSCs on the abluminal side [28]. With the structure of fenestrae, LSECs represent a permeable barrier allowing exchanges between two sides. However, LSECs do not simply form a barrier but exert instrumental effect in maintaining microenvironment homeostasis and mediating immune response in liver. Differentiated LSECs are the gatekeeper to maintain HSC in their quiescent state, which are critical to suppress fibrogenesis and carcinogenesis [29]. LSECs also influence the composition of hepatic immune populations by expression of adhesion molecules and chemokines [9]. The unique positioning, phenotype and function make LSECs an attractive candidate for organ-specific therapy. In our study, simvastatin exerted dual effect via LSECs to deactivate aHSCs and recruit NKT cells, leading to stromal regression and tumor suppression. Since LSECs are able to take up molecules by scavenger receptors, nanoparticles with target ligand can be an excellent way to apply therapy specific to LSECs. Several studies verified the therapeutic effect of nanoparticles targeting LSECs in various liver diseases [30–32]. Mannan is one of the most extensively used ligand to target mannose receptor on LSECs. In this study, PLGA with mannan ligand showed high liver accumulation and LSEC uptake rate, leading to satisfactory therapeutic effect in murine HCC model.

NKT cells which share phenotypic and functional features with NK cells represent a subpopulation of T lymphocytes. The liver is the organ with the highest amount of NKT cells compared with other T-cell subpopulations [33]. Indeed, NKT cells constitute about 30% of all lymphocytes in the liver, pointing to a critical role in liver disease [34]. Recently, several pieces of evidence showed that NKT cells exert important functions in antitumor immunity. It was revealed that hepatic NKT cell accumulation precedes HCC inhibition [35]. CXCR6-dependent Recruitment of NKT cells and CD4^+^ T cells to the liver exerts important functions in tumor surveillance to inhibit hepatocarcinogenesis [36]. Our findings demonstrated that the CXCL16 expression on LSECs induced by simvastatin attracted CXCR6^+^ NKT cells in the liver to inhibit tumor growth, which is consistent with previous studies. IFN-γ is instrumental for NKT initiated tumor immunity. Interestingly, it was found that NKT cells from cancer patients produce reduced amounts of IFN-γ than healthy subjects [37], while simvastatin targeted delivery to LSECs activated NKT cells and induced IFN-γ production, which contributed to anti-tumor effect.

## Conclusions

In summary, the therapeutic effect stems from the dual effect of simvastatin on tumor microenvironment in HCC, as well as the effective PLGA-PEG-MAN NP encapsulation and LSEC-targeted delivery. The LSEC-targeted delivery of simvastatin notably exerts effect on stromal microenvironment regression and NKT cell recruitment in fibrotic HCC mouse model. Besides, the lower dose than oral administration exerts higher efficiency with no toxicity, which would do great benefit to HCC patients in clinic whose liver function is vulnerable with limited therapeutic tolerability. Our findings reveal an immune-based therapeutic mechanism of simvastatin and offer a broad application of this drug to HCC patients based on tumor microenvironment remodeling.

## Supplementary Information

**Additional file 1: Figure S1**. Simvastatin shows no obvious cytotoxicity. Cytotoxicity study of SK-Hep1, LX2 and Huh7 cells treated with simvastatin at different concentrations (n = 3). **Figure S2.** Simvastatin inhibits the activity of HSC via LSEC. (A) The expression of KLF2 and eNOS was quantified in SK-Hep1 cells treated with indicated concentration of simvastatin for 24 h (n = 3). (B) The expression of α-SMA and collagen1 was quantified in LPS-activated LX2 treated with the supernatant of SK-Hep1 upon different doses of simvastatin treatment with or without L-NAME (n = 3). (C) The expression of CXCL16 was quantified in SK-Hep1 cells treated with the indicated concentrations of simvastatin for 24 h (n = 3). **p < 0.01, ***p < 0.001. **Figure S3**. Synthesis of the LSEC-targeting PLGA-PEG. (A) The synthesis process of PLGA-PEG-mannan. (B) ^1^H-NMR spectra of the synthesized particles PLGA-PEG-mannan. Comparing the integrated area of peak g (mannan protons) and peak a (protons in PEG), the ratio of mannose to PEG is approximately 4.5. **Figure S4.** Simvastatin NPs show no obvious toxicity in mice. (A) HE staining of major organs from mice with various treatments. Scale bar stands for 50 μm. (B) Body weight changes during treatment. (C) Mice blood routine test and (D) Hepatorenal function test after treatment (n = 5). n.s. = not significant. **Table S1.** Antibody list. **Table S2.** Gene Primer list for real-time PCR. **Table S3.** Cytokine Primer list for real-time PCR.

## Data Availability

All data generated or analyzed during this study are included in this published article and its additional files.

## Abbreviations

HCC: Hepatocellular carcinoma
LSECs: Liver sinusoidal endothelial cells
HSCs: Hepatic stellate cells
aHSCs: Activated hepatic stellate cells
HMG-CoA: 3-Hydroxy-3-methylglutaryl CoA
KLF2: Kruppel-like factor
NO: Nitric oxide
CXCL: Chemokine (C-X-C motif) ligand
CXCR: Chemokine (C-X-C motif) receptor
NKT: Natural killer T
PD-1: Programmed death receptor 1
PD-L1: Programmed death-1-ligand-1
NPs: Nanoparticles
PFA: Paraformaldehyde
H&E: Hematoxylin ad eosin
IHC: Immunohistochemistry
IF: Immunofluorescence
TUNEL: Terminal deoxynucleotidyltransferase-mediated dUTP nick end labeling
RT-PCR: Real-time polymerase chain reaction
DMSO: Dimethylsulfoxide
TEM: Transmission electron microscopy
EE: Encapsulation efficiency
LC: Loading capacity
AST: Aspartate aminotransferase
ALT: Alanine aminotransferase
BUN: Urea nitrogen
CRE: Creatinine
αSMA: α-Smooth muscle actin
LYVE1: Lymphatic vessel endothelial hyaluronan receptor 1
eNOS: Endothelial nitric oxide synthase
LPS: Lipopolysaccharide
RNA-seq: RNA-sequencing
PLGA: Poly(lactic-co-glycolic acid)
PEG: Polyethylene glycol
MAN: Mannan
