# Supplementary material for: Nano delivery of simvastatin targets liver sinusoidal endothelial cells to remodel tumor microenvironment for hepatocellular carcinoma
Source: J Nanobiotechnology. 2022 Jan 4;20:9. doi: 10.1186/s12951-021-01205-8 (PMC8725360; doi:10.1186/s12951-021-01205-8)
Supplement: Supplementary file 1 — Additional file 1: Figure S1. Simvastatin shows no obvious cytotoxicity. Cytotoxicity study of SK-Hep1, LX2 and Huh7 cells treated with simvastatin at different concentrations (n = 3). Figure S2. Simvastatin inhibits the activity of HSC via LSEC. (A) The expression of KLF2 and eNOS was quantified in SK-Hep1 cells treated with indicated concentration of simvastatin for 24 h (n = 3). (B) The expression of α-SMA and collagen1 was quantified in LPS-activated LX2 treated with the supernatant of SK-Hep1 upon different doses of simvastatin treatment with or without L-NAME (n = 3). (C) The expression of CXCL16 was quantified in SK-Hep1 cells treated with the indicated concentrations of simvastatin for 24 h (n = 3). **p < 0.01, ***p < 0.001. Figure S3. Synthesis of the LSEC-targeting PLGA-PEG. (A) The synthesis process of PLGA-PEG-mannan. (B) 1H-NMR spectra of the synthesized particles PLGA-PEG-mannan. Comparing the integrated area of peak g (mannan protons) and peak a (protons in PEG), the ratio of mannose to PEG is approximately 4.5. Figure S4. Simvastatin NPs show no obvious toxicity in mice. (A) HE staining of major organs from mice with various treatments. Scale bar stands for 50 μm. (B) Body weight changes during treatment. (C) Mice blood routine test and (D) Hepatorenal function test after treatment (n = 5). n.s. = not significant. Table S1. Antibody list. Table S2. Gene Primer list for real-time PCR. Table S3. Cytokine Primer list for real-time PCR. [file 12951_2021_1205_MOESM1_ESM.docx]

Additional file for

**Nano delivery of simvastatin targets liver sinusoidal endothelial cells to remodel tumor microenvironment for hepatocellular carcinoma**

Zhuo Yu^1,2^, Jianfeng Guo^1,3^, Yun Liu^1^, Menglin Wang^1^, Zhengsheng Liu^1^, Yueqiu Gao^2^*, Leaf Huang^1^*

^1^Division of Pharmacoengineering and Molecular Pharmaceutics, Eshelman School of Pharmacy, University of North Carolina, Chapel Hill, NC, 27599, USA.

^2^Department of Liver Disease, Shuguang Hospital, Affiliated to Shanghai University of Traditional Chinese Medicine, Shanghai 201203, China.

^3^School of Pharmaceutical Sciences, Jilin University, Changchun 130021, China.

***Correspondence authors: Leaf Huang** ([leafh@email.unc.edu](mailto:leafh@email.unc.edu));

**Yueqiu Gao** (gaoyueqiu@shutcm.edu.cn)


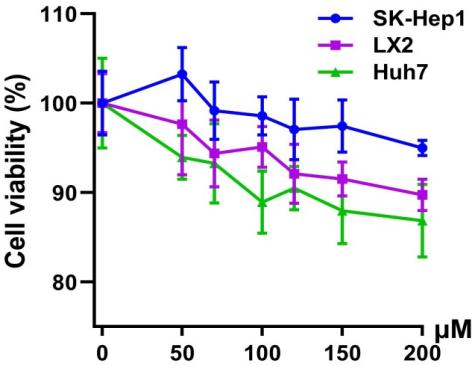


**Figure S1**. **Simvastatin shows no obvious cytotoxicity**. Cytotoxicity study of SK-Hep1, LX2 and Huh7 cells treated with simvastatin at different concentrations (n = 3).


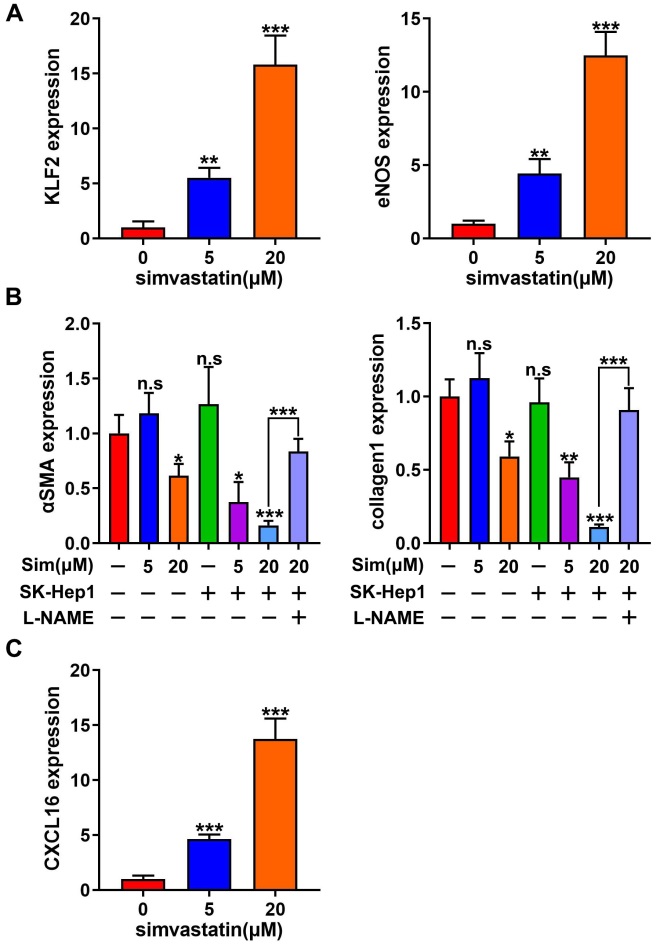


**Figure S2. Simvastatin inhibits the activity of HSC via LSEC.** (A) The expression of KLF2 and eNOS was quantified in SK-Hep1 cells treated with indicated concentration of simvastatin for 24 h (n=3). (B) The expression of α-SMA and collagen1 was quantified in LPS-activated LX2 treated with the supernatant of SK-Hep1 upon different doses of simvastatin treatment with or without L-NAME (n=3). (C) The expression of CXCL16 was quantified in SK-Hep1 cells treated with the indicated concentrations of simvastatin for 24 h (n=3). ***p* < 0.01, ****p* < 0.001.


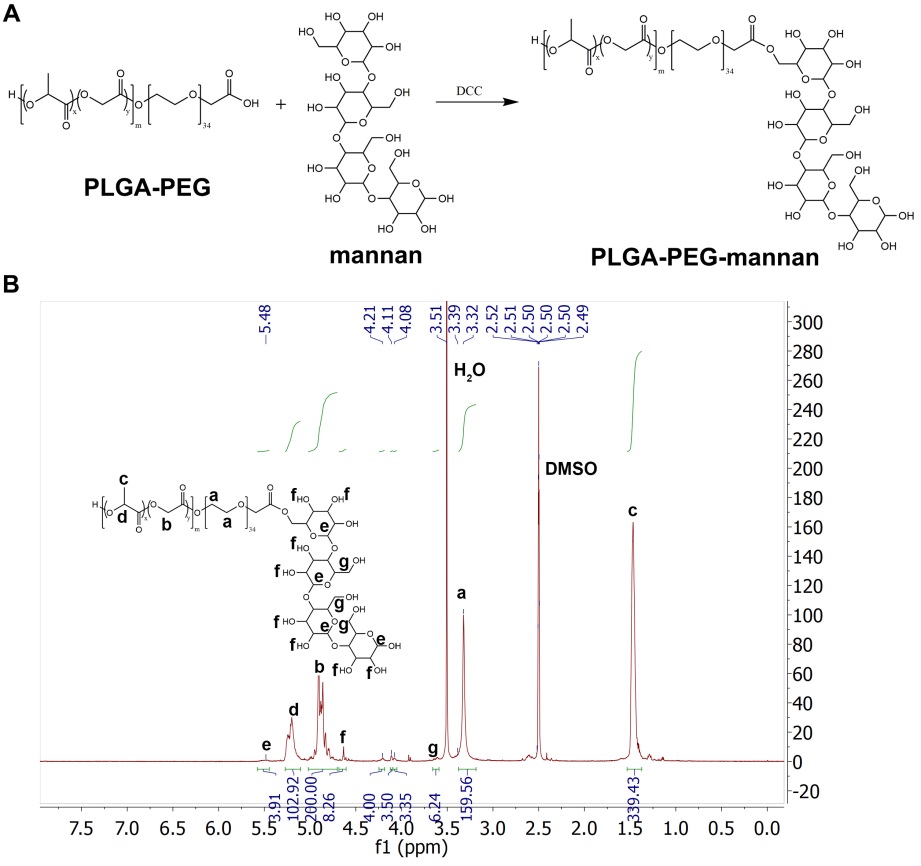


**Figure S3**. **Synthesis of the LSEC-targeting PLGA-PEG**. (A) The synthesis process of PLGA-PEG-mannan. (B) ^1^H-NMR spectra of the synthesized particles PLGA-PEG-mannan. Comparing the integrated area of peak g (mannan protons) and peak a (protons in PEG), the ratio of mannose to PEG is approximately 4.5.

**
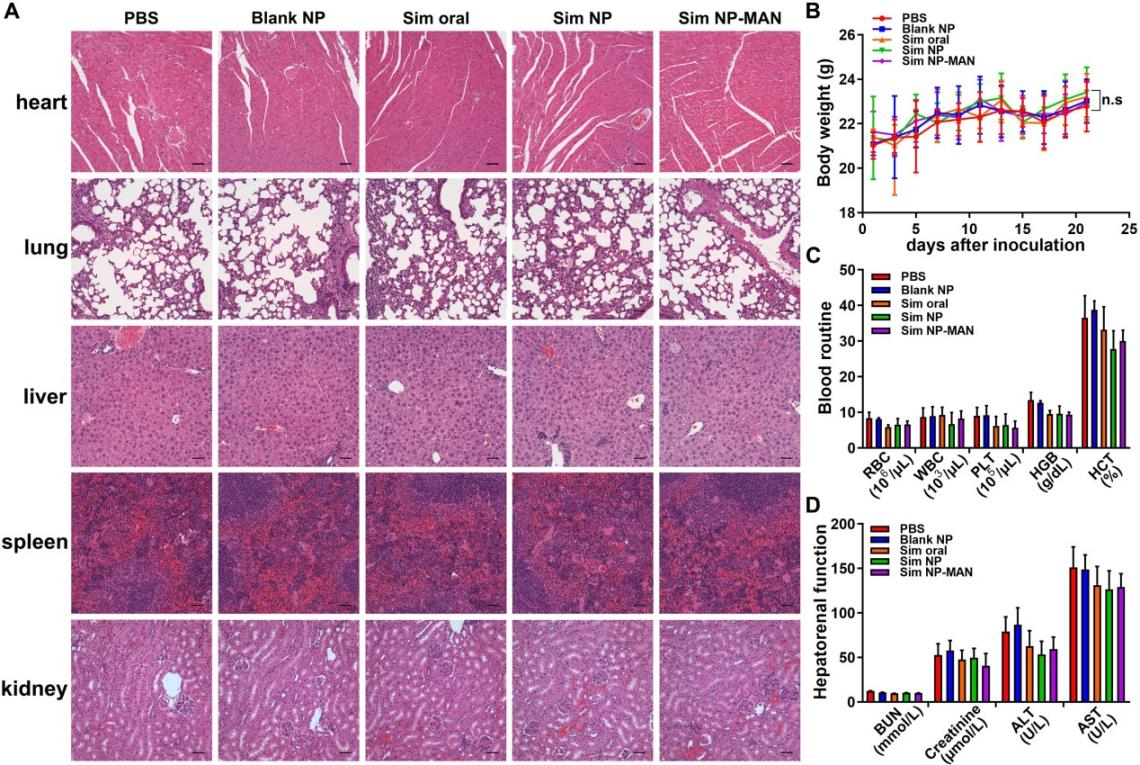
**

**Figure S4.** **Simvastatin NPs show no obvious toxicity in mice.** (A) HE staining of major organs from mice with various treatments. Scale bar stands for 50 μm. (B) Body weight changes during treatment. (C) Mice blood routine test and (D) Hepatorenal function test after treatment (n=5). n.s. = not significant.

**Table S1. Antibody list**

| Antibody | Company | Catalog | Application |
| --- | --- | --- | --- |
| Anti-KLF2 | Abcam | ab194486 | WB |
| Anti-eNOS | Abcam | ab199956 | WB |
| Anti-Collagen1 | Abcam | ab6308 | WB |
| Anti-GAPDH | Abcam | ab181602 | WB |
| Anti-CXCL16 | Abcam | ab101404 | WB |
| Anti-α-SMA | Abcam | ab7817 | IF, IHC, WB |
| Anti-CD31 | Abcam | ab28364 | IF, IHC |
| Anti-CXCL16 | Bioss antibodies | bs-1441R | IF |
| Anti-LYVE1  (Alexa Fluor® 488-conjugated) | eBioscience | 53044380 | IF |
| CD1d-tetramer (PE-conjugated) | Proimmune | - | FC |
| Anti-CXCR6 (FITC-conjugated) | BioLegend | 151107 | FC |
| Anti-CD69 (APC-conjugated) | BioLegend | 104513 | FC |
| Anti-IFNγ (APC-conjugated) | BioLegend | 505809 | FC |
| Anti-PDL1 (APC-conjugated) | BioLegend | 124311 | FC |

**Table S2. Gene Primer list for real-time PCR**

| Primer | | Sequence |
| --- | --- | --- |
| Mus musculus | *Klf2* | F: 5’-GAGCCTATCTTGCCGTCCTTT-3’’ |
|  |  | R: 5’-CACGTTGTTTAGGTCCTCATCC-3’ |
|  | *Nos3* | F: 5’-GGCTGGGTTTAGGGCTGTG-3’ |
|  |  | R: 5’-CTGAGGGTGTCGTAGGTGATG-3’ |
|  | *Gapdh* | F: 5’-TGGCCTTCCGTGTTCCTAC-3’ |
|  |  | R: 5’-GAGTTGCTGTTGAAGTCGCA-3’ |
| Human sapiens | *KLF2* | F: 5’-CTACACCAAGAGTTCGCATCTG-3’ |
|  |  | R: 5’-CCGTGTGCTTTCGGTAGTG-3’ |
|  | *NOS3* | F: 5’-TGATGGCGAAGCGAGTGAAG-3’ |
|  |  | R: 5’-ACTCATCCATACACAGGACCC-3’ |
|  | *ACTA2* | F:5’-CTATGAGGGCTATGCCTTGCC-3’ |
|  |  | R:5’-GCTCAGCAGTAGTAACGAAGGA-3’ |
|  | *COL1* | F:5’-GAGGGCCAAGACGAAGACATC-3’ |
|  |  | R:5’-CAGATCACGTCATCGCACAAC-3’ |
|  | *CXCL16* | F:5’-GACATGCTTACTCGGGGATTG-3’ |
|  |  | R:5’-GGACAGTGATCCTACTGGGAG-3’ |
|  | *GAPDH* | F:5’-GGAGCGAGATCCCTCCAAAAT-3’ |
|  |  | R:5’-GGCTGTTGTCATACTTCTCATGG-3’ |

**Table S3. Cytokine Primer list for real-time PCR**

| Primer (mouse) | Applied Biosystems/Ref |
| --- | --- |
| CXCL16 | Mm00469712_m1 |
| TGFβ | [Mm01298616_m1](https://www.thermofisher.com/taqman-gene-expression/product/Mm01298616_m1?CID=&ICID=&subtype=) |
| CXCL9 | Mm00434946_m1 |
| IL-6 | [Mm00446190_m1](https://www.thermofisher.com/taqman-gene-expression/product/Mm00446190_m1?CID=&ICID=&subtype=) |
| IFN-γ | Mm01168134_m1 |
| TNFα | Mm00443260_g1 |
| GAPDH | Mm99999915_g1 |
